# Myocardial T2 mapping is better than T2W Turbo Spin Echo for the diagnosis of acute myocarditis at 3T CMR

**DOI:** 10.1186/1532-429X-15-S1-P131

**Published:** 2013-01-30

**Authors:** Elsa Fernandes, Tamara Rothstein, Gabriel C Camargo, Daniel C Quintella, Maria Eduarda Derenne, Patricia B Rizzi, Ralph Strecker, Andreas Greiser, Peter Kellman, Joao A Lima, Ronaldo SL Lima, Ilan Gottlieb

**Affiliations:** 1 CDPI - Clínica de Diagnóstico por Imagem, Rio de Janeiro, Brazil; 2 Siemens Ltda, São Paulo, Brazil; 3 Laboratory of Cardiac Energetics, National Institutes of Health, Bethesda, MD, USA; 4 Medicine/Cardiology, Johns Hopkins University, Baltimore, MD, USA; 5 Siemens Healthcare, Erlangen, Germany

## Background

Myocardial edema is usually assessed by T2W Turbo Spin Echo (TSE), using either Triple Inversion Recovery (STIR) or Double Inversion Recovery (DIR) with spectral fat saturation. Both TSE sequences have important drawbacks, such as low SNR when using the body coil, dropout of the lateral wall signal and use of semi-quantitative analysis by computing the ratio of myocardial to skeletal muscle signal intensity. We aimed to test a newly developed SSFP-based bright blood sequence that uses surface coils and quantifies myocardial T2 times on a pixel-by-pixel basis (T2 mapping).

## Methods

**Figure 1:**
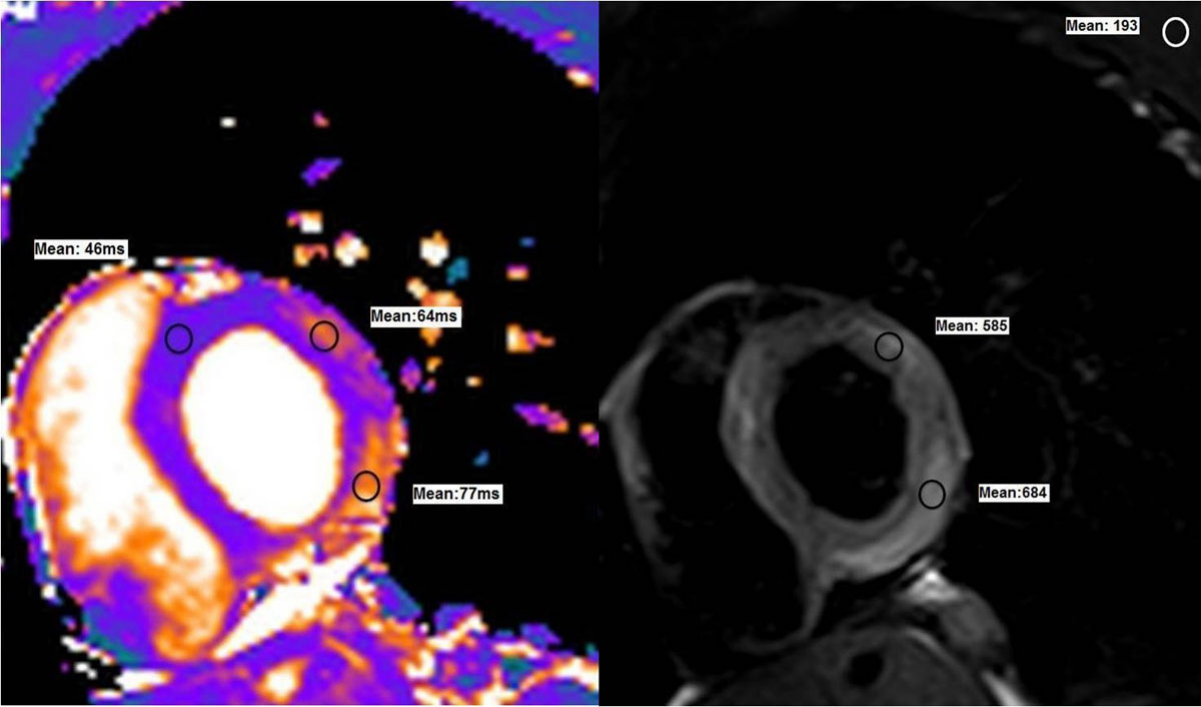
T2 map (left) and turbo spin echo (right) images of midventricular short axis view. Regions of interest drawn in the anterolateral and inferolateral walls are compared to remote myocardium (T2 map) and skeletal muscle (TSE) in order to assess the presence of edema in those areas.

Forty consecutive patients referred for CMR for acute myocarditis rule out within 15 days of symptoms onset were included. All patients were scanned in a 3T system (Verio, Siemens, Germany) with matched T2W TSE with spectral fat sat and body coil and T2 map sequences on three short axis slices (Figure 1). The final diagnosis of myocarditis was defined by consensus of two experienced cardiologists using clinical, ECG, laboratory and CMR information (for ischemic cardiomyopathy exclusion only). All analyses were blinded to the other modality's result and to clinical data. Image quality was assessed by segment using a scale from 0 (non-evaluable) to 3 (no artifacts). Area under the ROC curve was performed for accuracy assessment in a patient-based analysis and linear correlation was used for assessing the relationship between TSE and T2 map.

## Results

A total of 14 (36%) patients were clinically diagnosed as acute myocarditis. The relationship between TSE and T2 map was poor in both per segment and per patient analyses (R= 0.28 and 0.33, respectively). T2 map had higher diagnostic accuracy per patient than TSE, with an area under the ROC curve of 0.78 and 0.69, respectively, p<0.001 for the difference. Based on the ROC curve, the best threshold for T2 map was 52 msec with sensitivity of 79% and specificity of 68%. The best threshold for TSE ratio was 2,1 with sensitivity of 79% and specificity of 60%. Segment-based image quality was better in T2 map versus TSE, with the percentage of grades 0 or 1 (poor quality) of 4% and 20% respectively, p<0.001.

## Conclusions

Edema determination by T2 mapping has higher diagnostic accuracy for identifying patients with acute myocarditis than the usually performed T2W TSE. T2 map has also better overall image quality.

## Funding

Internal

